# Items, Selections and Remarks

**Published:** 1869-08

**Authors:** W. W. Miner


### Items, Selections and Remarks.

BY W. W. MINER, A. B.

Prof. Lister, of the University of Glasgow, has, in the June number of the *Lancet*, an article on the *Antiseptic system of Ligature.* He proposes to avoid suppuration of surgical wounds by means of an antiseptic bath, and, by using for ligatures, animal tissue that shall be absorbed, thus allowing immediate and complete cicatrization. He cites three experimental cases, in one of which he uses strips from the peritoneum of the small intestine of an ox, in place of the ordinary silken thread. A sound cicatrix was formed in about a week; and, at the end of a month, the greater portion of the ligature was found to be absorbed and replaced by living fibrous tissue. The knot contained fragments of the original peritoneal tissue in process of replacement, while a firm living fibrous cord, supplanting the original ligature, encircled the artery, and was continuous with its outer coat. He recommends a cord prepared from the small intestines of sheep, which has been treated with a solution of carbolic acid in five parts of olive oil, in which a little water is diffused. Great care is taken to destroy all germs of putrefaction, which might enter the wound, by the use of carbolic acid solution. Prof. Lister recommends "torsion" in the case of all the small arteries.

From fourteen of the medical colleges of the country, a total of 1008 medical students have graduated this year.——Prof. Benj. Howard's method of artificial respiration has been taught the police of New York city.——Dr. Flint's articles on the "Rochester Knockings," which first appeared in the *Buffalo Medical Journal*, are reproduced in the July number of the *Psychological Quarterly*.——The American Otological and Ophthalmological Societies held their annual meetings at Newport, commencing July 20th and continuing three days. The latter society now numbers sixty-one members.

The vapor of carbolic acid, obtained by heating a solution of the acid over a gas-jet, furnishes a very ready and effective means for fumigation. An advantage this vapor has over chlorine and sulphurous acid gases, is that it is not injurious to metalic surfaces.——Prof. Moxey, of England, administers food to insane persons, and those unable to take it in the natural way, by means of the nose. The patient is placed in a lying posture, and involuntarily swallows the liquid, which is introduced in suitable doses, through his nostril.——The *Reporter* says: "A citizen of Brunswick, Me., who earns his living working by the day, has paid for morphia, for the use of his wife, nearly thirteen hundred dollars, during the past fourteen years.——M. Kœberle, of Strasburg, has performed ovariotomy in twenty-five cases, from June 1st, 1868, to April 1st, 1869. Twenty of the patients have recovered and five have died.

The English Chemical Society has instituted a gold medal, in honor of the late Prof. Faraday, to be given to such foreign chemists as shall attain eminence in the science.——Geo. A. Peters, surgeon to the New York hospital, has an article on acupressure in the June number of the *N Y Medical Journal,* occupying thirty-six pages, and accompanied by sixteen illustrative wood-cuts. In the July number of the same Journal, Prof. Austin Flint has an excellent article on "Prognosis in Bright's Disease."——Dr. Magni, Prof. of Ophthalmology, in the University of Bologna, Italy, has gone to Lima, Peru, to operate on a merchant for cataract. Besides the expenses of the journey for himself and assistant, he is to be paid a fee of one thousand francs, about twenty thousand dollars in gold.

Ferric hydrate combines with cane sugar to form what is termed saccharated ferric hydrate. In this definite compound, saccharose seems to combine with iron very much as ammonium does with other metals. This substance may be precipiated with alcohol from its solution, washed, dried, powdered and preserved, without alteration. It combines ten per cent. of metallic iron, and dissolves in water without decomposition. It is of a brownish color, and has no styptic taste. Bonbons of this substance, containing one-fifteenth of a grain of iron, are in use in Germany. In addition to preserving the general therapeutic properties of iron in a very pleasant form, it constitutes the best antidote for arsenic. Arsenious acid easily decomposes it, liberating the sugar and forming an insoluble iron compound. The German journals give accounts of it, which are receiving considerable attention among pharmaceutists.

Union College, at its last commencement, conferred the degree of L. L. D. on Prof. Frank H. Hamilton.——Dr. Austin Flint, Jr., has received a prize from the French Academy of Science for his "Experimental Researches on a new Function of the Liver."——The "N. Y. City Dermatological Society," the first of its kind in the world, has just been established, with H. D. Buckley, as President; T. D. Wiesse, Vice-President; and Fred. Zinnser, as Corresponding Secretary.——The "Society for Reporting the Progress of Medicine," has been formed in New York City, consisting of one representative from each of the departments of medicine. The officers are H. Knapp, President; and A. L. Carroll, Secretary. Meetings are held on the second and fourth Thursdays in each month.

Prof. E Andrews, of Chicago Medical College, has recently tried Prof. Lister's System of Ligatures, and with good result.——Herr Strollberger, a silversmith of Munich, after various experiments, has found that, for protecting polished surfaces of silver from the blackening consequent on exposure, a coating of collodion is the most efficient and excellent means. He dilutes common collodion with alcohol and applies a very thin coating with a soft brush.——"Nelaton saved his first case of croup when tracheotomy was performed, and then operated twenty-three times without a recovery. Probably he operated, under all circumstances, like the majority of French Physicians."—*Medical Record.* Rather severe.

M. Tardieu has reported to the French Academy of Sciences, two cases of poisoning, caused by wearing stockings dyed with *coralline red.* This brilliant coloring substance, wherever brought in contact with the skin, caused blisters, some of which reached ulceration, while a general feeling of pain affected the system. Experiments on animals show that this fashionable dye is a very energetic poison. ——Saw-dust pills would effectually cure many of the diseases with which mankind is afflicted, if every individual would make his own saw-dust.—*Reporter.*

